# Radiomics and comparative neuroimaging of canine gliomas and human glioblastomas: a step toward precision medicine

**DOI:** 10.3389/fvets.2026.1893069

**Published:** 2026-09-01

**Authors:** Ricardo Faustino, Joaquim Henriques

**Affiliations:** 1 CrossI&D: Lisbon Research Center, Escola Superior de Saúde da Cruz Vermelha Portuguesa (ESSCVP), Lisbon, Portugal; 2 Instituto de Biofísica e Engenharia Biomédica, Faculdade de Ciências da Universidade de Lisboa, Lisbon, Portugal; 3 BioRG, Faculty of Engineering, Lusófona University, Lisbon, Portugal; 4 I-MVET, Faculty of Veterinary Medicine, Lusófona University, Lisbon, Portugal; 5 Anicura Atlantico Veterinary Hospital, Mafra, Portugal; 6 iNOVA4Health, IPO-Lisboa, Lisboa, Portugal

**Keywords:** comparative oncology, glioma, imaging biomarkers, magnetic resonance imaging, precision medicine, radiomics, reverse translational medicine

## Abstract

**Introduction:**

Gliomas are primary Central Nervous System glial tumors common in dogs and humans. Human glioblastomas are the most aggressive subtype, with an average survival of 15 months after diagnosis. Brachycephalic dogs are predisposed to gliomas and share biological and clinical features with human glioblastomas. This study explored radiomics applied to T2 weigthed Fluid Attenuated Inversion Recovery (T2-FLAIR) magnetic resonance imaging (MRI) sequences to characterize canine glioma lesions and non-lesioned tissues, aiming to identify imaging biomarkers for diagnosis and prognosis.

**Methods:**

MRI scans from canine gliomas and human glioblastomas were analyzed. Regions of Interest (ROIs) were segmented for extraction of radiomic features including texture, shape, and intensity. Descriptive and non-parametric statistical analyses, ANOVA, and Receiver Operating Characteristic (ROC) curves were used to evaluate feature discrimination capacity, particularly features with area under the curve (AUC) values above 80%, such as “jointaverage” and “autocorrelation”.

**Results:**

Volumetric analysis revealed significant differences between groups (*p* < 0.001). Selected radiomic features discriminated tumoral from non-lesioned tissues, with ROC AUC values exceeding 80% (*p* < 0.001). The Support Vector Machine (SVM) model achieved 80.33% accuracy in distinguishing gliomas and glioblastomas from non-lesioned brain tissue across canine and human datasets.

**Discussion:**

Radiomic features revealed comparable texture patterns between canine gliomas and human glioblastomas, enabling cross-species discrimination between tumoral and non-lesioned tissue with AUC values above 80% and SVM accuracy of 80.33%. These findings support canine gliomas as a valuable translational model for human glioblastoma research and precision medicine. However, the limited sample size and population diversity require validation in larger cohorts.

**Conclusion:**

Consistent imaging biomarkers across canine gliomas and human glioblastomas support advances in comparative and translational medicine. Radiomic features extracted from T2-FLAIR MRI enabled non-invasive discrimination between tumoral and non-lesioned brain tissue across species, reinforcing the relevance of canine gliomas as a natural model for human glioblastoma and the potential of comparative neuroimaging for precision medicine.

## 1 Introduction

Gliomas are primary central nervous system (CNS) tumors that can affect both humans and dogs, significantly impacting health and quality of life. Glioblastoma (GBM) is the most aggressive and common malignant glial cell tumor in humans, accounting for 48.6% of intracranial malignancies. It is characterized by rapid growth, significant biological heterogeneity, and resistance to conventional treatments, resulting in a poor prognosis and an average survival of 15 months after diagnosis (1–4). In dogs, gliomas account for 36%−70% of intracranial neoplasms (5), with higher prevalence in brachycephalic breeds such as Boxers and Bulldogs. These tumors share biological features with human GBMs, including infiltrative growth and molecular complexity (6). Genetic studies have identified molecular variants such as CAMKK2 and P2RX7 associated with tumor development, indicating predisposition and potential therapeutic targets (7, 8). Treatments such as surgery and chemotherapy with temozolomide show limited effectiveness due to the tumour's infiltrative nature and high recurrence rates (9–11). These similarities position canine gliomas as valuable natural models for comparative oncology and neuroimaging studies, reinforcing the “One Medicine” concept (12).

Magnetic resonance imaging (MRI) is the primary imaging modality for characterizing gliomas in both species (13). Sequences such as T2-FLAIR are particularly effective in visualizing lesions near cerebrospinal fluid interfaces (14), frequently revealing heterogeneous intensity and peritumoral edema, which are useful for prognostic evaluation (5). In humans, advanced techniques such as perfusion imaging, namely Dynamic Susceptibility Contrast Magnetic Resonance Imaging (DSC-MRI), diffusion (DWI), Magnetic Resonance Spectroscopy (MRS), and functional Magnetic Resonance Imaging (fMRI) are employed to assess vascularization, white matter tract integrity, and tumor metabolism, respectively. In dogs, the use of these techniques is more limited, but conventional protocols (T1, T2, FLAIR, Gradient-Recalled-Echo GRE) already provide essential information on edema, mass effect, and invasion patterns (15–18).

Radiomics, an emerging field in diagnostic imaging, transforms medical images into quantitative data, enabling advanced analyses with machine learning (19). The concept of radiomics has evolved considerably over the past decade, following the pioneering work of Kumar et al. (20), Lambin et al. (21), and Aerts et al. (22), who established the extraction of quantitative imaging biomarkers as a promising approach for tumor characterization, prognosis, and precision medicine. This approach is particularly valuable for assessing tumor heterogeneity, an important factor in predicting tumor behavior and treatment resistance (23). In veterinary medicine, radiomics faces challenges such as variability in acquisition protocols and image segmentation, but its potential for identifying biomarkers is undeniable (24). Human studies have already correlated radiomic features, such as texture and shape, with clinical outcomes, and similar advances are expected to benefit veterinary practices (25).

The integration of comparative neuroimaging with radiomics strengthens translational research. Comparative neuroimaging studies have shown that human and canine brains share important neuroanatomical structures, while also exhibiting species-specific functional organization. Functional differences, such as olfactory specialization in dogs and greater planning capacity in the human frontal lobe, are also evident (26, 27). These anatomical and functional differences impact tumor response patterns, emphasizing the importance of species-specific approaches (28).

From a therapeutic perspective, the Stupp protocol (surgery, radiotherapy, and temozolomide) is the standard in humans but offers limited benefits due to the nearly universal recurrence of GBMs (4, 29, 30). In dogs, treatment options include surgery and chemotherapy, with temozolomide and lomustine demonstrating the ability to cross the blood-brain barrier and slow tumor progression (9, 11, 31). In humans, phenomena such as pseudoprogression, associated with MGMT methylation, pose challenges in differential diagnosis even with advanced MRI techniques (32).

Under the umbrella of One Health-One Medicine context, the development of comparative imaging can help find biomarkers for personalized oncology strategies, in both humans and dogs. Despite technical barriers such as standardization and automated segmentation, the integration of radiomics and artificial intelligence promises to improve diagnosis and prognosis in both species, aligning with a truly translational approach (33, 34).

In this study we aimed to identify shared radiomic biomarkers that reflect the biological and structural characteristics of gliomas across species. By bridging veterinary and human oncology, this study contributes to the understanding of glioma heterogeneity and supports the development of non-invasive diagnostic tools.

## 2 Materials and methods

### 2.1 Patients

For this study, 39 series of images from 29 dogs, previously diagnosed with glioma and 16 from humans diagnosed with glioblastomas) were selected from a public database. The series comprised a volumetric analysis of T1-weighted images due to their superior differentiation of gray matter (GM) and white matter (WM). Of the 23 canine series, only 16 were retained for radiomic analysis of T2-FLAIR images (the remaining were excluded due to non-cerebral studies or lack of necessary T2-FLAIR images). In total, 31 patients were selected (dogs, *n* = 15; humans, *n* = 16) for data extraction and radiomic analysis (Tables 1–3).

**Table 1:** Demographic data of the canine patient for radiomic analysis.

Breed	*n* (%)	Sex	*n* (%)	Mean age (standard deviation)
Boxer	4 (26.67)	Male	10 (66.67)	8.57 (2.17)
Boston Terrier	6 (40)	Female	5 (33.33)	8.86 (2.20)
Labrador Retriever	2 (13.33)			
Mixed Breed	2 (13.33)			
Yorkshire Terrier	1 (6.67)			
Total	15 (100)		15 (100)	8.73 (2.7)

**Table 2:** Demographic data of the canine patient for volumetric analysis.

Raça	*n* (%)	Sex	*n* (%)	Mean age (standard deviation)
Boxer	6 (26.11)	Male	15 (65)	8.32 (2.34)
Boston terrier	7 (30.43)	Female	8 (35)	8.48 (2.44)
Labrador retriever	2 (8.7)			
Mixed breed	4 (17.4)			
Yorkshire terrier	1 (4.34)			
French bulldog	1 (4.34)			
Staffordshire bull terrier	1 (4.34)			
Bulldog	1 (4.34)			
Total	23 (100)		23 (100)	8.43 (2.36)

**Table 3:** Demographic data of the human sample for radiomic analysis.

Gender	*n* (%)	Mean age (standard deviation)	Days of survival after surgery
Male	12 (75)	57 (12.38)	756 (615.59)
Female	4 (25)	54.03 (12.08)	832.55 (678.64)
Total	16 (100)	56.97(11.69)	740.44(589.45)

Both datasets were obtained from The Cancer Imaging Archive (TCIA): the ICDC-Glioma collection (36) provided by the Frederick National Laboratory for Cancer Research, consisting of data from 81 dogs diagnosed with glioma, and the UPenn-GBM collection, consisting of data from 630 human glioblastoma patients from the University of Pennsylvania (37).

The inclusion criteria for both data collections were the presence of histopathological confirmation of the tumor type, as well as the assurance of MRI image quality, particularly T2-FLAIR-weighted images, which are fundamental for accurate segmentation of the regions of interest and essential for radiomic feature extraction and analysis.

### 2.2 MRI sequences for volumetry and radiomic characterization

**Figure 1:**
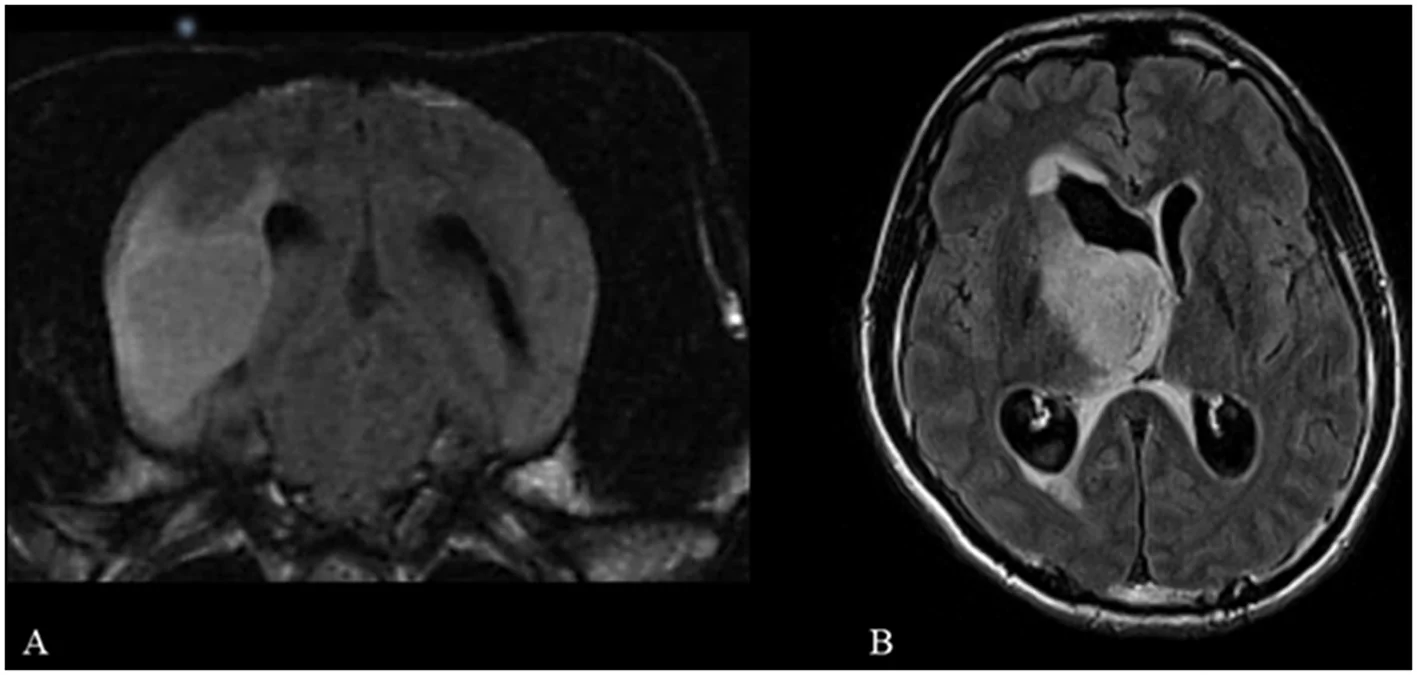
**(A)** Axial slice of a canine brain with an extensive tumor lesion, showing characteristic hyperintensity in T2-FLAIR, located in the right cerebral hemisphere, emphasizing the similarities with the corresponding axial slice in **(B)** Of a patient with human glioblastoma characterized in the present study.

Among the various MRI sequences, T2-FLAIR images were used due to their suppression of cerebrospinal fluid (CSF) signal, which increases contrast between lesion areas and surrounding brain tissue. This sequence allows visualization of edema and tumor infiltration associated with increased water content resulting from vasogenic edema (38). For this reason, T2-FLAIR images were selected for tumor delineation in canine gliomas and human glioblastomas, supporting segmentation procedures and subsequent radiomic feature extraction (Figure 1).

### 2.3 Segmentation and volumetric analysis

MRI images in DICOM (Digital Imaging and Communications in Medicine) format were uploaded to the Syngo. Via workstation (https://www.siemens-healthineers.com/magnetic-resonance-imaging/clinical-specialities/neuro-mr-imaging), where the dedicated MR Neurology workflow was used for visualization and post-processing of neuroimaging data. In this initial phase, all exams were reviewed for a preliminary neuroradiological evaluation to ensure data integrity, particularly the absence of artifacts that could compromise image quality.

The previously published methodology facilitated segmentation, volume measurement, radiomic feature extraction, and analysis using 3DSlicer, an open-source platform for medical image processing and analysis (39).

Segmentation of the various anatomical structures of interest was performed using the Segment Editor module available in 3DSlicer. The segmentation of gliomas and glioblastomas was carried out on each MRI slice using a semi-automatic segmentation approach (40) based on the Thresholding and Grow from Seeds tools, which provided greater precision in delineating the regions of interest ROIs. The imaging characteristics of hyperintense areas (tumor tissue and edema) on T2-FLAIR-weighted images facilitated the distinction between areas with different tissue characteristics.

The accuracy of the segmentations was assessed through visual inspection of the ROIs in three planes: axial, sagittal, and dorsal for canine data, and axial, sagittal, and coronal for human T2-FLAIR images. Manual adjustments were made as needed to optimize ROI delineation, ensuring the consistency of the segmentations.

The automatic calculation of the volume of interest (VOI) for each tumor lesion was performed using the Segment Statistics module. Volumes, measured in milliliters (ml), were based on the number and size of voxels within each ROI. In addition to quantifying the extent of the tumor lesion in each brain, this methodology was also applied to canine brain volumetry. For the human MRI examinations, skull stripping was performed using the HD Brain Extraction Tool (HD-BET), which has been developed and validated for human brain MRI. Because no validated implementation is currently available for canine neuroimaging (41), HD-BET was not applied to the canine dataset in order to avoid using the algorithm outside its validated domain.

### 2.4 Radiomics features extraction and analysis

The lesion volumes obtained through segmentation were used to create a 3D model to enable the extraction of radiomic features contained within the VOIs, using the SlicerRadiomics extension from the Pyradiomics library (35). Pyradiomics is an open-source Python package used to calculate a total of 69 radiomic features (All information available on pyradiomics documentation (https://pyradiomics.readthedocs.io/en/latest/), structured into the following classes: first-order statistics (19 features), shape-based features (26 features), and gray-level co-occurrence matrix (GLCM) features (24 features) (53). In addition to tumor lesions, radiomic features were also extracted from VOIs of “normal” brain tissue, i.e., areas without apparent lesions, typically in the contralateral hemisphere.

Radiomic features were extracted directly from the native image geometry without isotropic voxel resampling. Since this retrospective study relied on publicly available MRI datasets with different acquisition characteristics, preserving the original image geometry avoided introducing additional interpolation-related variability into the extracted texture features.

The feature extraction was performed using the default preprocessing settings implemented in the SlicerRadiomics/PyRadiomics workflow, with image intensities discretized using a fixed bin width of 25 intensity units, corresponding to the default PyRadiomics configuration (https://pyradiomics.readthedocs.io/en/v3.0/customization.html?).

The methods used in this study followed the radiomic approach proposed by Faustino et al., (39). Radiomic analysis of the VOIs derived from the medical images focused primarily on the extraction of first-order features and GLCM-derived features (42). For first-order features, fundamental statistics such as mean, median, standard deviation, minimum, and maximum were calculated. These parameters provide a broader understanding of the overall distribution of voxel intensities in the analyzed medical images. Additionally, GLCM-based features were used to capture specific spatial patterns and explore the relationships between intensity levels of voxel pairs.

Subsequently, comparative analysis focused on the classification between “tumor tissue” and “non-lesioned tissue,” as well as the evaluation of differences between species (dogs and humans). For this purpose, ROC curves were employed to determine sensitivity, specificity, and area under the curve (AUC) for each radiomic feature, enabling assessment of the classification power of each metric, i.e., its diagnostic accuracy. Comparison criteria included *p*-values < 0.05 to test statistical significance and the Youden index to evaluate the balance between sensitivity and specificity values for each radiomic feature.

### 2.5 Statistical analysis

Statistical analysis for the characterization and comparison of demographic data (canine and human) and radiomic features was performed using the Statistical Package for Social Sciences (IBM Corp., Released 2022. IBM SPSS Statistics for Windows, Version 29.0. Armonk, NY: IBM Corp.). The non-parametric Mann-Whitney test was used for the comparison of quantitative data.

A repeated measures ANOVA was conducted to identify significant differences between the “tumor tissue” and “tissue without lesions” groups within subjects. Results with *p*-values < 0.05 were considered statistically significant.

The data were subjected to a principal component analysis (PCA) for dimensionality reduction and selection of the most relevant variables. This analytical method transforms the original variables into a new set of variables, called principal components, providing a more compact and interpretable representation of the data. Each principal component is a weighted linear combination of the original features, representing latent patterns in radiomic characteristics that best explain the variance in the data.

Subsequently, ROC curves were calculated to assess the performance of radiomic features in discriminating between different tissue types, considering sensitivity, specificity, and the Youden index.

The results of the comparative analysis demonstrated that radiomic features such as “Autocorrelation” and “JointAverage” had AUC values exceeding 87%, underscoring their potential as biomarkers for distinguishing between tumor and non-tumor tissue. Significant differences were observed in features associated with heterogeneity (e.g., “JointEntropy” and “SumEntropy”), suggesting variations in the aggressiveness and complexity of the analyzed tumors. These findings will be presented and discussed in the results sections.

### 2.6 Development of an artificial intelligence model

For the classification of ROIs as tumor tissue or non-lesioned tissue, an artificial intelligence (AI) model based on Machine Learning (ML) was developed using a Support Vector Machine (SVM) classifier in MATLAB R2022a. The MathWorks, Inc., Natick, Massachusetts, United States, 2022 (https://www.mathworks.com/products/new_products/release2022a.html).

To minimize the risk of overfitting, only the radiomic features selected through the PCA and ROC analyses were included in the SVM classifier, thereby reducing model complexity relative to the available dataset.

The dataset was divided into two subsets: 70% of the data was used for training and validation of the model, while the remaining 30% was used for testing. Subsequently, the model was evaluated using a confusion matrix, which allowed for the calculation of accuracy and the identification of true positives (TP), true negatives (TN), false positives (FP), and false negatives (FN). Finally, the trained model was applied to the entire dataset to assess its overall performance.

## 3 Results

### 3.1 Volumetry analysis

The results of the volumetric analysis are presented in Table 4. This analysis enabled the determination of tumor and brain volumes (ml). To facilitate comparisons between species and among patients within the same sample with varying brain volumes, a normalization of values was performed to calculate the volume difference in both species using the following formula:


$$\begin{array}{c} Volume\;difference=\frac{Brain\;volume-Total\;tumor\;volume}{Brain\;volume} \end{array}$$ (1)

**Figure 2:**
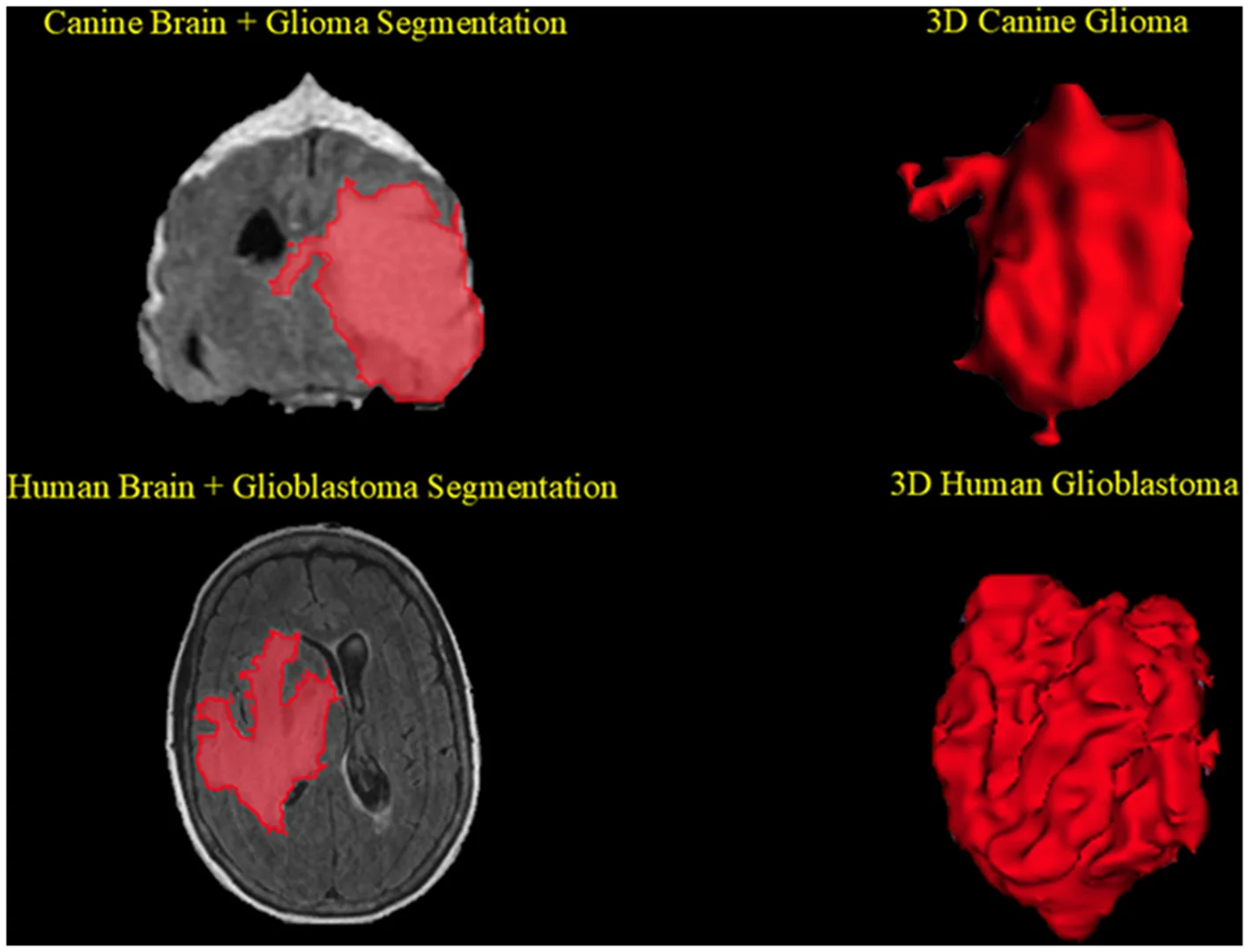
The segmentation of tumor lesions on T2-FLAIR-weighted images (in axial view in this figure) is a fundamental step for volumetric analysis as well as for subsequent extraction of radiomic features.

This normalization enables comparisons between species with varying brain volumes, eliminating the impact of absolute brain size. The volume difference calculates the fraction of brain volume not occupied by the tumor lesion. A value approaching 1 indicates that the tumor occupies a smaller portion of the brain, while the inverse suggests a larger relative occupation. For a more direct comparison, the respective ratio of brain volume occupied by the tumor, expressed as a percentage, was calculated as a complementary metric following tumor lesion segmentation (Figure 2).

**Table 4:** Volumetric results for the canine and human patients.

Dogs	*n*	Min	Max	Mean	SD
Tumor volume (ml)	23	2.34	18.93	9.83	4.61
Brain volume (ml)	23	39.10	112.52	93.91	16.64
Volume difference	23	0.81	0,97	0.89	0.05
Rate (%)	23	2.85	18.62	10.54	4.52
Humans					
Tumor volume (ml)	16	8.86	195.05	81.19	49.78
Brain volume (ml)	16	1,307.29	1,763.36	1,527.24	125.92
Volume difference	16	0.86	0.99	0.95	0.03
Rate (%)	16	0.61	13.89	5.29	3.35

The Mann–Whitney statistical test demonstrated statistically significant differences (*p* < 0.001) between the volumetric data of dogs and humans for all variables

### 3.2 Radiomics analysis results

The normality tests, particularly the Kolmogorov-Smirnov test, demonstrated that the majority of the extracted radiomic features do not follow a normal distribution (with the exception of the SumEntropy feature, which showed a *p*-value > 0.005). This supports the use of non-parametric tests, such as the Mann-Whitney test, for comparing distributions.

Of the 69 radiomic features extracted, only 10 were ultimately selected following the PCA and ROC analysis.

The PCA analysis identified the features that were most effective in detecting patterns related to tumor tissue (gliomas and glioblastomas). The first three principal components had eigenvalues of 21.98, 12.71, and 6.45, respectively. These values account for 68.56% of the cumulative variance.

The communality values of the variables, exceeding 0.9, indicate that a significant portion of the original variance was retained in the principal components. This result confirms the efficiency of PCA in reducing the dimensionality of the data while preserving the key characteristics relevant to this study. The PCA results demonstrate that the first three principal components are essential for explaining the variability in the data.

To verify whether the selected radiomic features “JointEntropy,” “SumEntropy”, “SumAverage”, “JointAverage”, “SumSquares”, and “Autocorrelation” exhibited texture differences between tumor tissue and non-lesioned tissue, a Mann-Whitney test was performed, yielding a *p*-value < 0.001 for the various radiomic features, demonstrating statistically significant differences. These results were further corroborated by an ANOVA analysis, which also demonstrated the differences between tumor and non-lesioned tissue.

These findings underscore the importance of the selected radiomic features and their potential to discriminate between neoplastic and non-lesioned tissues, with a *p*-value < 0.001 and robust eta-squared values (η^2^) (ranging between 0.271 and 0.384), reinforcing the clinical relevance of the observed variations (see Table 5).

**Table 5:** Analysis of radiomic features with repeated measures ANOVA.

Radiomic features	*Z* (between groups)	η2	Tissue type	*p*
SumAverage	36.752	0.384	Lesion vs. Without Lesion	< 0.001
SumEntropy	33.900	0.365	Lesion vs. Without Lesion	< 0.001
SumSquares	21.953	0.271	Lesion vs. Without Lesion	< 0.001
JointEntropy	20.353	0.256	Lesion vs. Without Lesion	< 0.001
JointAverage	36.752	0.384	Lesion vs. Without Lesion	< 0.001
Autocorrelation	31.183	0.346	Lesion vs. Without Lesion	< 0.001

Results from a repeated measures analysis of variance (ANOVA) for various radiomic features (GLCM) across different tissue groups, including tumoral lesions and tissue without lesions, are presented. The *Z*-values (between groups), effect sizes (η^2^), and *p*-values are reported for each radiomic feature, emphasizing significant differences in mean values among the groups. Bonferroni-adjusted multiple comparisons were applied.

The Mann-Whitney test for species analysis revealed that similar texture patterns exist, with no statistically significant differences (*p* > 0.05). However, some features, such as “JointEntropy” and “SumSquares”, showed greater variation in canine gliomas compared to human glioblastomas, with *p*-values of 0.395 and 0.171, respectively.

**Figure 3:**
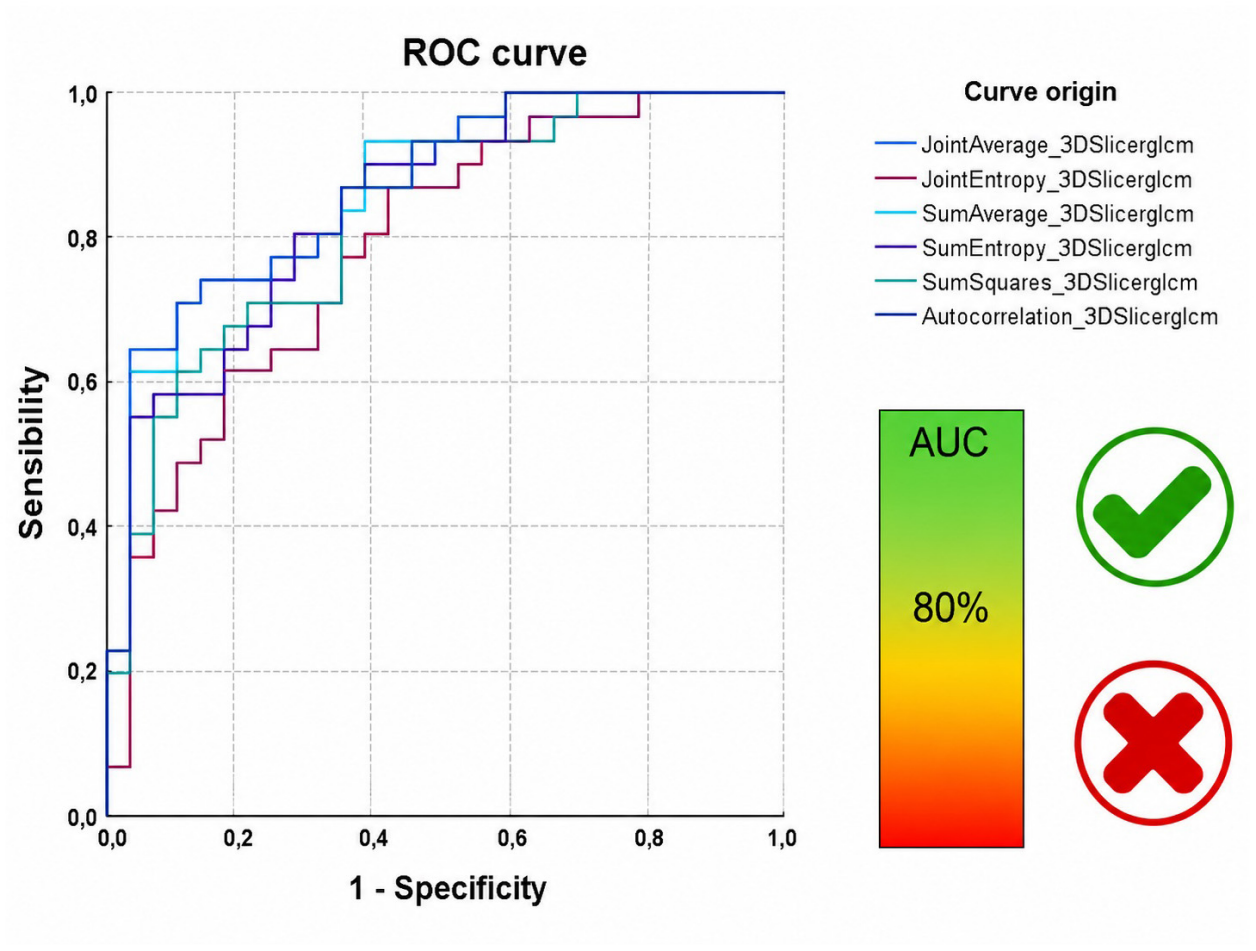
ROC Curves after the selection of radiomic features, emphasizing the three main features with AUC Values > 80%: “JointAverage”, “SumAverage”, and “Autocorrelation”, Respectively (*p* < 0.001).

To evaluate the discriminatory performance of the selected radiomic features for classifying regions of interest (ROIs) as tumor lesion or non-lesioned tissue, receiver operating characteristic (ROC) analysis was performed. The six best-performing GLCM features achieved AUC values ranging from 0.796 to 0.874, all with statistically significant discriminatory performance (*p* < 0.001), which remained significant after Benjamini–Hochberg false discovery rate (FDR) correction (adjusted *p* < 0.001). Among these, Autocorrelation showed the highest diagnostic performance (AUC = 0.874; 95% CI: 0.788–0.960), with a sensitivity of 65% and a specificity of 97%, while JointAverage and SumAverage also demonstrated excellent discrimination (AUC = 0.871) (Table 6; Figure 3).

**Table 6:** ROC curve results.

Radiomic features	AUC(95%CI)	Sensibility	Specificity	YImax	*p*	Adjusted *p* (FDR)
JointAverage	0.870 (0.784–0.958)	0.770	0.730	0.507	< 0.001	< 0.001
JointEntropy	0.800 (0.684–0.907)	0.770	0.670	0.441	< 0.001	< 0.001
SumAverage	0.870 (0.784–0.958)	0.710	0.900	0.610	< 0.001	< 0.001
SumEntropy	0.850 (0.760–0.945)	0.810	0.730	0.539	< 0.001	< 0.001
SumSquares	0.840 (0.743–0.939)	0.870	0.670	0.538	< 0.001	< 0.001
Autocorrelation	0.870 (0.788–0.960)	0.650	0.970	0.612	< 0.001	< 0.001

Receiver operating characteristic (ROC) analysis of the six best-performing GLCM radiomic features. Area under the curve (AUC) values with 95% confidence intervals (95% CI), sensitivity, specificity, and the corresponding maximum Youden Index (YImax) are reported. Statistical significance was assessed for each ROC curve, and *p*-values were adjusted for multiple comparisons using the Benjamini–Hochberg false discovery rate (FDR) procedure.

### 3.3 Machine learning—support vector machine results

**Figure 4:**
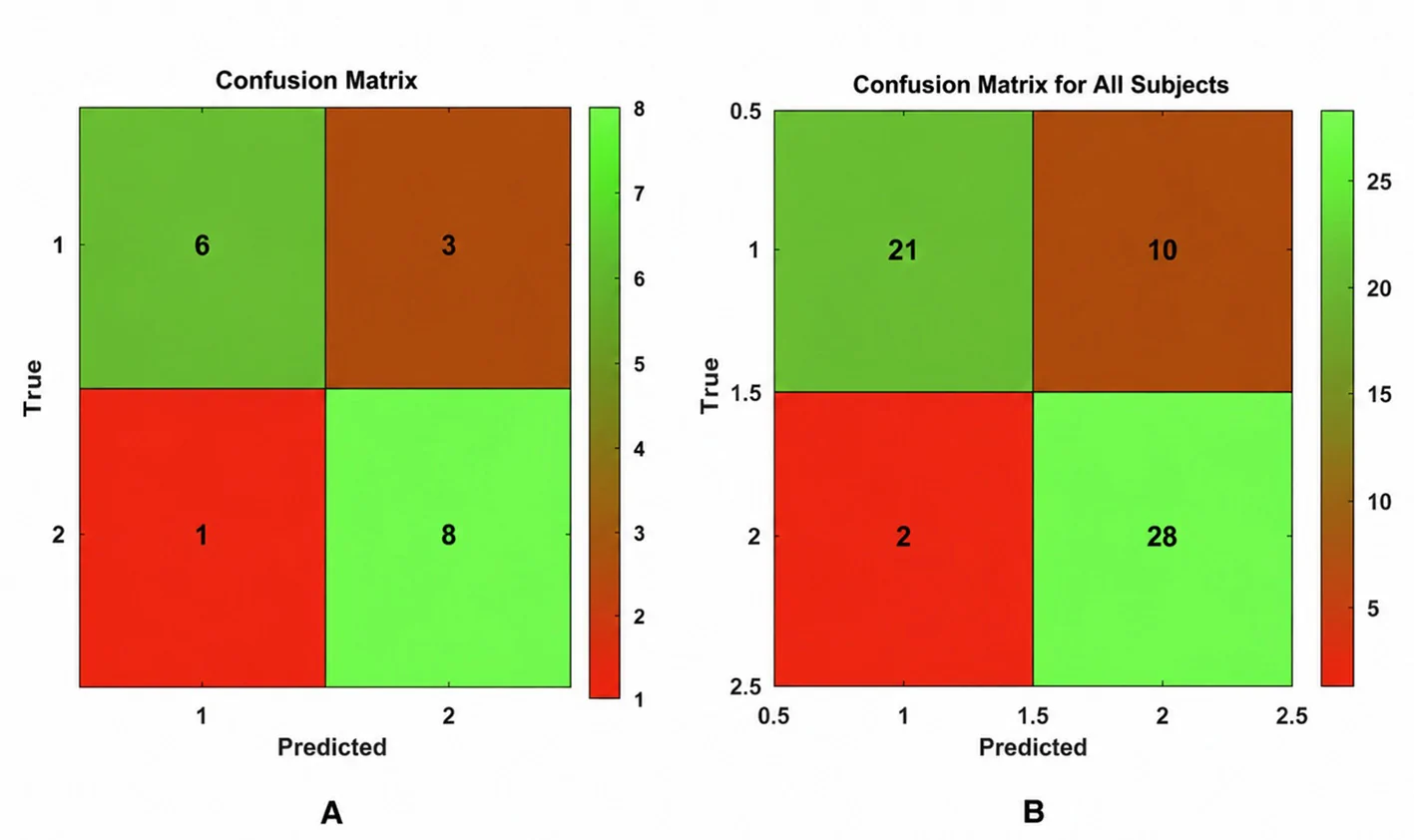
**(A)** Confusion matrix for the test group, showing the following results: 6 true positives (TP), 8 true negatives (TN), 1 false positive (FP), and 3 false negatives (FN). The model achieved a classification accuracy of 77.88% for this dataset. **(B)** Confusion matrix for the total patient group, showing the following results: 21 true positives (TP), 28 true negatives (TN), 2 false positives (FP), and 10 false negatives (FN). The model achieved a classification accuracy of 80.33% for this dataset.

Considering the promising results described above, an AI model based on an SVM classifier was developed. Its evaluation for the test group resulted in a confusion matrix with the following outcomes: 6 true positives (TP), 8 true negatives (TN), 1 false positive (FP), and 3 false negatives (FN). The model achieved a classification accuracy of 77.88% for the test group, demonstrating promising performance in classifying ROIs as tumor or non-lesioned tissue (Figure 4).

After the training phase, the model was tested on a group comprising the entire dataset. The confusion matrix yielded the following results: 21 true positives (TP), 28 true negatives (TN), 2 false positives (FP), and 10 false negatives (FN). The model achieved a classification accuracy of 80.33% for the total dataset, confirming its robustness when generalized to the entire dataset (Figure 4).

## 4 Discussion

### 4.1 Influence of brain volume differences between species

The volumetric analysis results in dogs showed that tumor lesions occupy an average of 10.5% of total brain volume, while in humans, tumor lesions occupy an average of 5.29%. Although the normalized brain volume differences between dogs and humans are smaller, the ratios demonstrate a larger brain volume in humans. In both cases, the Mann-Whitney test demonstrated statistically significant differences (*p* < 0.001) for all volumetric variables. These findings emphasize the importance of normalization procedures in comparative neuroimaging studies, as substantial differences in overall brain volume between species can strongly influence volumetric interpretations. Beyond global measures of tumor burden, brain tumors may also be associated with more subtle and distributed morphological changes in brain structure. In this context, voxel-based morphometry (VBM) approaches, widely applied in the study of neurodegenerative diseases, have also demonstrated relevance in neuro-oncology by enabling the assessment of regional structural variations beyond simple volumetric metrics (43, 44).

### 4.2 Tumor heterogeneity and malignancy

The results of this study provide evidence that the extracted radiomic features can discriminate between tumor and non-lesioned tissues in both canine gliomas and human glioblastomas. Radiomic features extracted from T2-FLAIR MRI images using the GLCM method showed potential as imaging biomarkers for assessing tumor heterogeneity, uniformity, and tissue complexity (see Table 7).

**Table 7:** Definition of radiomic features and their biological interpretation.

Radiomic features	Radiomics defintion	Biological interpretation	Reported biological associations
JointAverage	Mean of combined intensities of neighboring voxels.	Tumor uniformity and average texture, reflecting the degree of homogeneity within the tumor tissue.	Reported association with homogeneous receptor and structural protein distribution and relatively organized PI3K/Akt and MAPK signaling.
JointEntropy	Complexity and disorder in combined intensities.	>Cellular heterogeneity and variability in tissue composition. More aggressive and less organized biology.	Reported association with increased cellular heterogeneity and differential activation of molecular pathways, including EGFR and apoptosis-related signaling (p53/Bcl-2)
SumAverage	Mean of the sums of voxel values in pairs.	Consistency of the tumor texture and tissue uniformity, reflecting the distribution of tumor cells and overall tissue density.	Reported association with a more uniform expression of signaling molecules and a relatively controlled tumor microenvironment.
SumEntropy	Disorder of the sums of voxel values.	Complex and heterogeneous tumor structure	Reported association with heterogeneous activation of signaling pathways, including Wnt/β-catenin and Notch.
SumSquares	Variation of voxel values relative to the mean.	Significant intra-tumoral heterogeneity, necrosis, and tumor aggressiveness.	Reported association with increased intratumoral heterogeneity and differential activation of pathways such as HIF-1α and VEGF.
Autocorrelation	Similarity between voxel values at specific distances.	Regular and homogeneous texture pattern within the tumor tissue.	Reported association with relatively homogeneous receptor/ligand expression and coordinated signaling involving TGF-β.

The proposed biological associations are based on previously published radiogenomic and molecular imaging studies, including Fathi Kazerooni et al. (46), Beig et al. (51), Guan et al. (45), and related literature. These associations are intended as hypothesis-generating interpretations rather than direct molecular evidence from the present study.

High intratumoral heterogeneity is associated with more aggressive tumor biology and a poor prognosis in human glioblastomas (19, 45). In this study, radiomic features such as “JointEntropy” and “SumEntropy”, reflecting voxel intensity complexity and disorder, showed statistically significant differences (*p* < 0.001) between tumor and non-lesioned tissue ROIs. These results suggest greater cellular and structural variability in lesioned compared to non-lesioned brain regions, aligning with literature describing more aggressive biology in gliomas with high heterogeneity (46, 47).

When comparing canine gliomas and human glioblastomas, radiomic features showed no statistically significant differences, indicating similar patterns of heterogeneity. This similarity provides preliminary evidence supporting the hypothesis that canine gliomas may represent a valuable translational model for human glioblastoma research. However, given the limited sample size of the present study, these findings should be interpreted cautiously and require confirmation in larger independent cohorts.

The present findings are consistent with recent MRI-based radiomics studies demonstrating that quantitative texture features can capture clinically and biologically relevant aspects of glioma heterogeneity. Fathi Kazerooni et al. (48) reported that radiomic SVM models derived from multiparametric MRI achieved moderate discriminatory performance for overall survival risk stratification in glioblastoma, with AUC values ranging from 0.63 to 0.78 across development and independent replication cohorts. Similarly, Li et al. (49) demonstrated that T2-weighted MRI-derived radiomic signatures retained prognostic and biological value across independent and prospective glioma cohorts. Pease et al. (50) further showed that MRI radiomic features could predict clinically relevant molecular alterations, including EGFR amplification and MGMT promoter methylation, highlighting the potential of radiomics to provide non-invasive insights into tumor biology. Although the endpoints of these studies differ from those of the present comparative analysis, their findings support the ability of MRI-derived texture features to provide non-invasive information on tumor heterogeneity and biological phenotype.

### 4.3 Uniformity and cellular density

Radiomic features such as “JointAverage”, “SumAverage”, and “Autocorrelation”, associated with tissue texture uniformity and consistency, demonstrated discriminatory ability between tissue types. ROC analysis showed these features achieved an AUC of 87%, with sensitivity ranging from 65% to 77% and specificity from 73% to 97%, suggesting that tissue uniformity is a key marker for differentiating neoplastic from non-neoplastic tissues.

The “Autocorrelation” feature achieved the highest AUC (87%) and a specificity of 97%, indicating its efficacy in identifying neoplastic tissue with regular and homogeneous texture. This suggests that gliomas with less structural heterogeneity and more uniform cell distribution may correlate with less aggressive biology (51). In humans, peri-tumoral areas showed a positive correlation with angiogenesis pathways in males (Laws energy feature), suggesting that our autocorrelation radiomic feature may be involved in the same pathway (51).

### 4.4 Species differences and the relevance of canine gliomas as a translational model

One of the study's main objectives was to evaluate whether canine gliomas serve as a reliable natural model for studying human glioblastomas. The results indicate no substantial differences in radiomic features between the species, with similar patterns of heterogeneity and uniformity. These findings support the hypothesis that canine gliomas may serve as a promising radiomic translational model for human glioblastoma. Nevertheless, because this is an exploratory study based on a relatively small cohort, broader cross-species generalization requires validation in larger independent datasets.

However, the variability in heterogeneity metrics, such as “SumEntropy” and “JointEntropy”, in canine gliomas may reflect genetic and biological diversity between dog breeds, particularly brachycephalic breeds, which are more frequently affected by gliomas (12).

### 4.5 Clinical implications and future applications in personalized medicine

The development of imaging biomarkers to precisely differentiate tumoral from non-tumoral tissue has direct implications for precision medicine. Radiomics could play a significant role in glioma management, but its application has not yet been integrated into clinical decision-making (46). Radiomic features such as “JointAverage”, “SumAverage”, and “Autocorrelation” may contribute to future approaches for risk stratification, surgical planning, and treatment response evaluation when integrated with clinical, molecular and imaging data. The different sensitivity and specificity profiles observed among these radiomic features may also support different clinical applications. Features with higher sensitivity, such as “JointAverage”, may be more suitable for screening purposes, whereas highly specific features such as “Autocorrelation” may be more useful as confirmatory biomarkers. “SumAverage” demonstrated a more balanced diagnostic profile, suggesting potential utility in both scenarios. However, these potential applications were not evaluated in the present study and require validation in appropriately designed radiogenomic and prospective clinical studies.

Although these results are encouraging, the machine learning analysis presented here should be regarded as exploratory. External validation in larger independent cohorts, together with integration of clinical and molecular biomarkers, is required before these imaging biomarkers or the proposed classifier can be considered for clinical implementation.

### 4.6 Importance of developing an AI model

The SVM model emphasized the importance of the selected radiomic features for differentiating ROIs between tumor and non-lesioned tissue. With an accuracy of 77.88% in the test group and 80.33% in the total dataset, the model demonstrated consistent performance. Importantly, the classifier was developed using tissue ROIs from both canine gliomas and human glioblastomas without incorporating species as an input variable, indicating that the selected radiomic features retained their discriminatory capability across both species. Features related to tumor uniformity and heterogeneity, such as SumAverage, SumSquares, and Autocorrelation, proved fundamental for tumor texture characterization.

AI integration opens new horizons for clinical and preclinical applications, particularly when combined with non-invasive methodologies such as radiomic analysis of MRI images (52).

Overall, the SVM analysis complements the statistical findings by showing that the selected radiomic features retain discriminatory value when analyzed in combination, supporting the integration of radiomics and machine learning in comparative neuroimaging research. These findings further support the translational potential of the canine glioma model by demonstrating that common radiomic characteristics can discriminate tumor from non-lesioned tissue independently of species.

### 4.7 Limitations of the study and future work

Several limitations must be considered in this study. The sample size may affect the generalizability of the results. Additionally, variability in MRI neuroimaging acquisition protocols could introduce biases that impact the reproducibility of the findings. Furthermore, as tumor segmentation was performed using a standardized semi-automatic protocol rather than manual freehand delineation, segmentation reproducibility was not formally assessed using repeated independent delineations. HD-BET preprocessing was applied only to the human MRI examinations because the algorithm has been validated exclusively for human brain MRI; therefore, this methodological asymmetry should be considered when interpreting the results. Future studies should aim to increase sample size and standardize imaging acquisition protocols or use datasets with stable acquisition protocols to validate the identified biomarkers.

Beyond these methodological considerations, future research should also focus on integrating complementary biological data to improve the biological interpretation and clinical relevance of radiomic biomarkers. Conducting studies with multi-omics analyses, such as incorporating genomic and proteomic data alongside radiomics, could provide a more comprehensive characterization of tumor biology. Although the AI model demonstrated positive results, with approximately 80% accuracy, it emphasizes the need to integrate multiple levels of data to improve the precision of predictive models.

## 5 Conclusion

This study demonstrated the utility of radiomic analysis and AI models based on SVM, achieving an 80% discrimination accuracy between tumor and non-lesioned tissues in canine gliomas and human glioblastomas using features extracted from T2-FLAIR images. Metrics such as “JointEntropy”, “SumEntropy”, “JointAverage”, “SumAverage”, “SumSquares”, and Autocorrelation were effective in identifying texture patterns associated with tumor heterogeneity and uniformity. The area under the curve (AUC), particularly for features like Autocorrelation with a value of 87% and specificity of 97%, confirms the relevance of these imaging biomarkers for assessing tissue complexity and uniformity.

The comparison between species revealed radiomic similarities, suggesting that canine gliomas may represent a promising translational model for human glioblastoma. Nevertheless, these findings should be considered preliminary and require validation in larger independent cohorts before broader cross-species or clinical conclusions can be drawn.

## Data Availability

The original contributions presented in the study are included in the article/supplementary material, further inquiries can be directed to the corresponding author.
